# Density Estimation Based on Mixtures of Gaussians for Perovskite Solar Cells Modeling

**DOI:** 10.1021/acs.jcim.5c02017

**Published:** 2026-01-19

**Authors:** F. Alexander Sepúlveda, Daniel Cerro-Ramos, T. Jesper Jacobsson

**Affiliations:** † School of Electrical, Electronics and Telecommunications Engineering, 28014 Universidad Industrial de Santander, 680006 Bucaramanga, Colombia; ‡ Department of Physics, Chemistry and Biology (IFM), Linköping University, SE-581 83 Linköping, Sweden

## Abstract

Accurately modeling the complex relationships among synthesis parameters, material compositions, and performance metrics is essential for accelerating the development of perovskite solar cells (PSCs). In this context, machine learning (ML) has proven to be a valuable tool. While most ML applications in PSC research rely on discriminative “black-box” models, this study adopts a generative approach by modeling the joint probability density function. We employ Gaussian Mixture Models (GMMs), a pragmatic and interpretable choice well-suited for the scarce, low-dimensional tabular data typical of PSC research. This single GMM framework is evaluated on five distinct tasks: discovering clusters, regression, generating novel configurations, training on data sets with missing data and, inverse design of the experimental (synthesis) conditions. That is, assuming we have the perovskite material composition and a target PCE, we infer the experimental conditions. For this latter task we use a novel “GMM-Assisted Optimization” method, which demonstrates to be more effective than standard random-start optimization, achieving an RMSE of 1.52 against target PCEs, more than halving the 3.32 RMSE of the baseline. These findings highlight the power of probabilistic modeling for data-driven discovery in PSC research.

## Graphical abstract

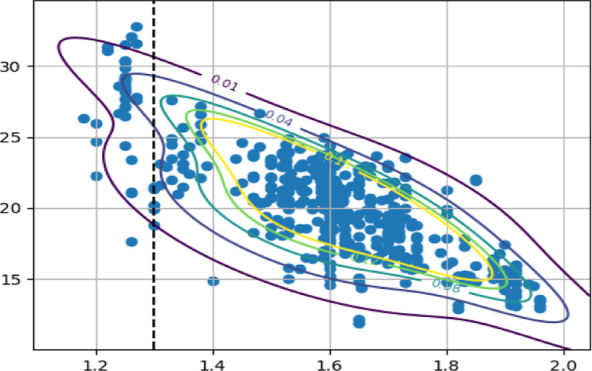

## Introduction

Over the past 15 years, hybrid perovskites have emerged as an exciting class of materials for a variety of optoelectronic applications, such as solar cells,^1^ light-emitting diodes,^2^ lasers,^3^ and X-ray detectors.^4^ Most of the focus has been given to solar cells (PSC), and the current certified efficiency record for single junction cells is currently at 27%[Fn fn1]. If challenges related to long-term stability and scalability can be addressed without sacrificing efficiency, perovskite solar cells have the potential to become a serious contender in the PV-market, especially in the form of tandem cells.^5–9^

As the field has grown, so has the interest in applying machine learning (ML) methods to model and optimize perovskite solar.^10^ In most cases, the ML methods used try to map from input descriptors *X*, such as synthetic conditions or elemental composition, to desired outputs *Y* such as band gap, device efficiency, or operational stability. In particular, Shrivastav et al.^11^ utilized the XGBoost algorithm, trained on simulated data obtained from SCAPS, to predict the performance of PSC.

A more interesting approach leverages experimental PSC synthesis data to train ML models. Training machine learning models requires high-quality data that accurately represents the population under analysis, both in terms of quality and quantity. Typically, more data improves model accuracy. The literature provides a wealth of valuable information about the synthesis and performance of perovskite solar cells and, given the recent surge in publications,^12^ a substantial amount of data has been produced. However, much of this information is unstructured and a significant effort is required to extract and consolidate it cohesively. This process can be approached manually;^13–16^ or be automated by using natural language processing techniques.^17^ As a result, perovskite data sets of varying scope and sizes are now available for training machine learning models.

In the context of PSC, machine learning-based tasks can be categorized as forward prediction, data generation, and inverse design. Forward prediction, the most common task, trains a model to predict device performance (the output) based on given material compositions and synthesis parameters (the inputs). Data generation is a process that uses a learned probability density function (PDF) to create new, synthetic-but-plausible experimental data containing the “recipes” and their corresponding performances. Finally, inverse design reverses the process of forward prediction. It searches the inputs (material compositions and synthesis parameters) that produce the target performance.

### Forward Prediction and Inference

Recently, several machine learning techniques have been employed to model and predict the behavior of PSCs. Among the most widely used methods are^18,19^ Linear Regression (LR) and Decision Trees (DT), which are inherently interpretable. Ensemble methods such as Random Forest (RF) and Extreme Gradient Boosting (XGBoost) have also gained traction due to their accuracy, robustness and effectiveness in handling high-dimensional data. Support Vector Machines (SVM) and Neural Networks (NN) are also popular. The authors in (Lu et al., 2023)^20^ (using a data set of 1072 devices extracted from peer-reviewed publications) reported XGBoost as the best model for PCE prediction compared to LR, NN, RF and GBDT (gradient boosting decision tree), wheras others have achived best results using RF–models (Chen et al., 2023).^10^ It is important to take into account that small data sets could influence the accuracy of ML predictions such that the results depend more on the quantity and representativeness of the data than on the specific modeling technique; i.e., the data quantity and quality could have a greater impact on predictive performance than the choice of algorithm itself. This is supported by the findings reported in (Mayr and Gagliardi, 2021)^21^ for the particular case of PSCs.

Currently, the most comprehensive data set on perovskite devices comes from the Perovskite Database Project^16,22^ that contains device-level data for over 42,000 metal-halide perovskite solar cells extracted from the peer-reviewed literature.^16^ This open-access resource enables large-scale, data-driven analyses and has recently been used in several projects, mainly for tasks related to inference and prediction of PSCs performance,^23–32^ but also for analyzing stability.^33,34^ In particular, to identify high-performance PSCs: Ping et al. (2025)^30^ employed a hierarchical machine learning approach—combining K-Means clustering with Histogram Gradient Boosting Trees (HGBT)—to detect devices with power conversion efficiency (PCE) exceeding 17%, which is a classification problem. Similarly, Hu et al. (2024)^24^ evaluated several ensemble learning algorithms, with the Voting Classifier (*V*_ot_) method achieving the best performance for identifying devices with PCE above 18%. Kusuma et al. (2025)^35^ developed predictive models for both PCE and device stability using RF and XGBoost. (Mammeri et al., 2023)^23^ also used RF.

Using the same data set,^16^ several studies have analyzed the importance of descriptors in respect to PCE. These works generally employ two types of metrics: model-dependent measures, such as SHAP (SHapley Additive exPlanations),^24,30,31,33,36^ and model-agnostic statistical measures, which depend solely on the data. The latter category includes Pearson correlation,^24,30,31,37^ Spearman correlation,^30^ and mutual information.^32^ Unlike standard correlations, this latter measure is particularly valuable as it can detect arbitrary nonlinear statistical associations.

### Generative Models

We could use the joint probability distribution as the model, instead of modeling only the conditional expectation,^38^ as in forward prediction algorithms. These models are called generative models^38,39^ due to their ability to generate new samples resembling the original they were trained on. Unlike forward prediction algorithms, the use of generative models for perovskite solar cells has been scarcely reported in the state of the art. The authors in (Chenebuah et al., 2024)^40^ propose a Lattice-Constrained Materials Generative Model, based on Variational Autoencoders (VAE) and Generative Adversarial Networks (GAN), for designing polymorphic perovskite materials with crystal conformities that are consistent with geometrical and thermodynamic stability constraints. The resulting material candidates were validated using Density Functional Theory (DFT). In contrast, (Iranipour et al., 2025)^41^ generated synthetic data using autoencoder and CGAN networks, which were then incorporated into a small existing data set from research articles. This technique improves the learning accuracy of deep learning models.

### Inverse Design

According to a recent review (Wang et al., 2022),^42^ inverse design methods can be divided into three main groups. The first, named High-Throughput Virtual Screening (HTVS), is a computer search through large, existing material data sets to identify candidates that meet certain performance ranges. The second, which we call optimization-based methods, uses metaheuristic algorithms like Genetic Algorithms (GA) for exploring the massive chemical space or Bayesian Optimization (BO) to smartly search for the best solution. Lastly, the third group, Generative Models (GM), uses models that can make new material structures that are not already in any database but fulfill a given condition.

So far, only a few studies have used the inverse design to speed up the search for new PSCs. One study^43^ utilized a Tandem Neural Network (TNN) architecture that learns to directly map a desired PCE with the target optical architecture. However, this model was trained on a large numerical simulation data set, which may not be able to capture the intricacies of making things in the real world. Another study proposed a method called Proactive Searching Progress (PSP), which uses a machine learning model to look through a huge virtual chemical space for the best Pb-free candidates. This model was trained on a database of more than 1200 real experimental band gaps taken from the literature.^44^ Bayesian Optimization (BO) is another very promising approach that is used to run a “closed-loop” experimental workflow.^45^ The main goal is not to learn the whole inverse mapping but to find the best-performing material by smartly and sequentially choosing the fewest real-world experiments to run. This sequential, data-efficient method is excellent for optimizing complicated materials when tests take a long time and are costly.

### Proposed Work

In this work, we propose a unified probabilistic framework for analyzing perovskite solar cell data based on a Gaussian Mixture Model (GMM). While various generative models exist, GMMs are a pragmatic and interpretable choice for our low-to-medium dimensional tabular data, which is expensive to acquire and thus limited in size. GMMs also provide direct insights into the data’s hidden cluster structure. Most importantly, GMMs can compute conditional probabilities, a property we leverage for generative inverse synthesis modeling. We apply this probabilistic framework to five distinct tasks: discovering clusters, regression, generating novel configurations, training on data sets with missing data (a common issue in experimental perovskite data sets), and solving the inverse problem of inferring experimental conditions from a target PCE. All tasks were carried out using real experimental data.

Regarding inverse synthesis design, we propose a two-stage hybrid method that strategically combines generative and optimization-based approaches. This approach is designed to navigate the “many-to-one” mapping problem in materials science. For our optimization, the forward mapping is represented by an XGBoost model, which, as a tree-based ensemble, does not provide analytical derivatives, thus requiring a derivative-free optimization algorithm. Our hybrid method overcomes this weakness by first using the GMM’s conditional probability to identify high-probability starting regions (“exploration”), and then an optimization procedure is applied.

## Method

### Data

In this work, we have used the open-access data set from the Perovsktie Database Project. (2022),^16^ which contains information on more than 42,400 perovskite solar cell devices. This information, collected from peer-reviewed scientific journals, include article metadata, device stack, perovskite composition, synthesis methodes, synthesis parameters, and key performance metrics.

The Perovskite Database is a good proof of concept data set due to the large number of observations and wide range of variables included. With this extensive data set, it becomes feasible to train machine learning (ML) models to predict performance metrics, such as Power Conversion Efficiency (PCE). This capability is particularly important as it enables the screening, or optimization, of various parameters. Furthermore, a trained model offers an interesting avenue for learning more about the underlying physics of these devices by inspecting the model’s behavior and performing feature importance techniques.

### Perovskite Material Representation

To apply machine learning algorithms, it is necessary to represent the materials as fixed-length vectors. In this work we have focused on the perovksite material and have not taken into account the hole transport materials, the electron transport materials, or any of the other materials in the device stack. A standard perovskite is an ABX_3_ structure, where each position can accommodate different ions, or combination of ions. The *X* site is occupied by a metal cation, typically Pb, Sn or a combination of Pb and Sn. The *X* site is occupied by a halide, such as Br, I, or Cl. The A-site contains a lot of variability and is occupied either by Cs or a small organic cation. The most common cations are methylammonium, MA and formamidinium, FA but a few hundred different cations have been reported in the literature.^46,47^ A simple method to represent the perovskite composition is shown in ElemNet,^48^ where the input vector includes values greater than zero for all the elements/ions in the compound and zero for those not present, i.e. a one hot encoding, followed by normalizing so that all the values add up to one. Using the raw proportions of these elements or compounds as input features presents two main challenges. First, it leads to unnecessarily high dimensionality, significantly increasing the number of model parameters and raising the risk of overfitting as well as issues associated with the curse of dimensionality. Second, the generated vectors are sparse.

In addition, the probabilistic distributions of these features are found to deviate significantly from the normal distribution. However, to model these complex distributions, Gaussian Mixture Models (GMMs) can be employed under the assumption that the data can be approximated as a combination of normal-like behaviors. Furthermore, having a low-dimensional space helps mitigate the curse of dimensionality when estimating a p.d.f. The goal in this step is to represent the perovskite material by dense vectors instead of sparse ones.

To address these challenges, we propose the use of Local Linear Embeddings (LLE). In particular, we propose a combination of ElemNet, that generates a vector of dimension 133 (the number of basic elements/ions that appear in the data set), with LLE. First, one-hot vectors are created for elements belonging to the perovskite material; then, sum-pooling is applied. Later, component-wise addition of the element vectors is perfored for the elements in the chemical formula. That is, for a perovskite compound whose formula includes *m* constituent elements (*v* ∈ *V*) the compound vector is given by

1 $$q=\sum_{k=1}^{m}c_{k}v_{k}$$

where *v*_k_ is the corresponding element vector for the *k*th constituent element in the formula, and *c*_k_ is the relative quantity of the *k*th constituent element in the compound. A total of 1837 unique vectors of dimension 133 are obtained.

To this point, we have a set of vectors of dimension 133 representing the material. We then employed Locally Linear Embedding (LLE) with the cosine distance to reduce this dimensionality. To identify the optimal intrinsic dimensionality, we performed a 5-fold cross-validation. This test revealed that a 4-dimensional manifold consistently preserved over 95% of the “local neighborhood trustworthiness” across all folds. Therefore, we proceeded with an LLE-transformed space of four components for our analysis. Next, we optimized the “number of neighbors” parameter, ultimately selecting 60 (60). In addition, this technique offers the advantage of not only reducing the dimensionality, but we also obtain statistical properties more similar to normal distributions, which are better suited for some machine learning techniques. Figure 1 shows the distributions of the 4 LLE components.

**Figure 1:**
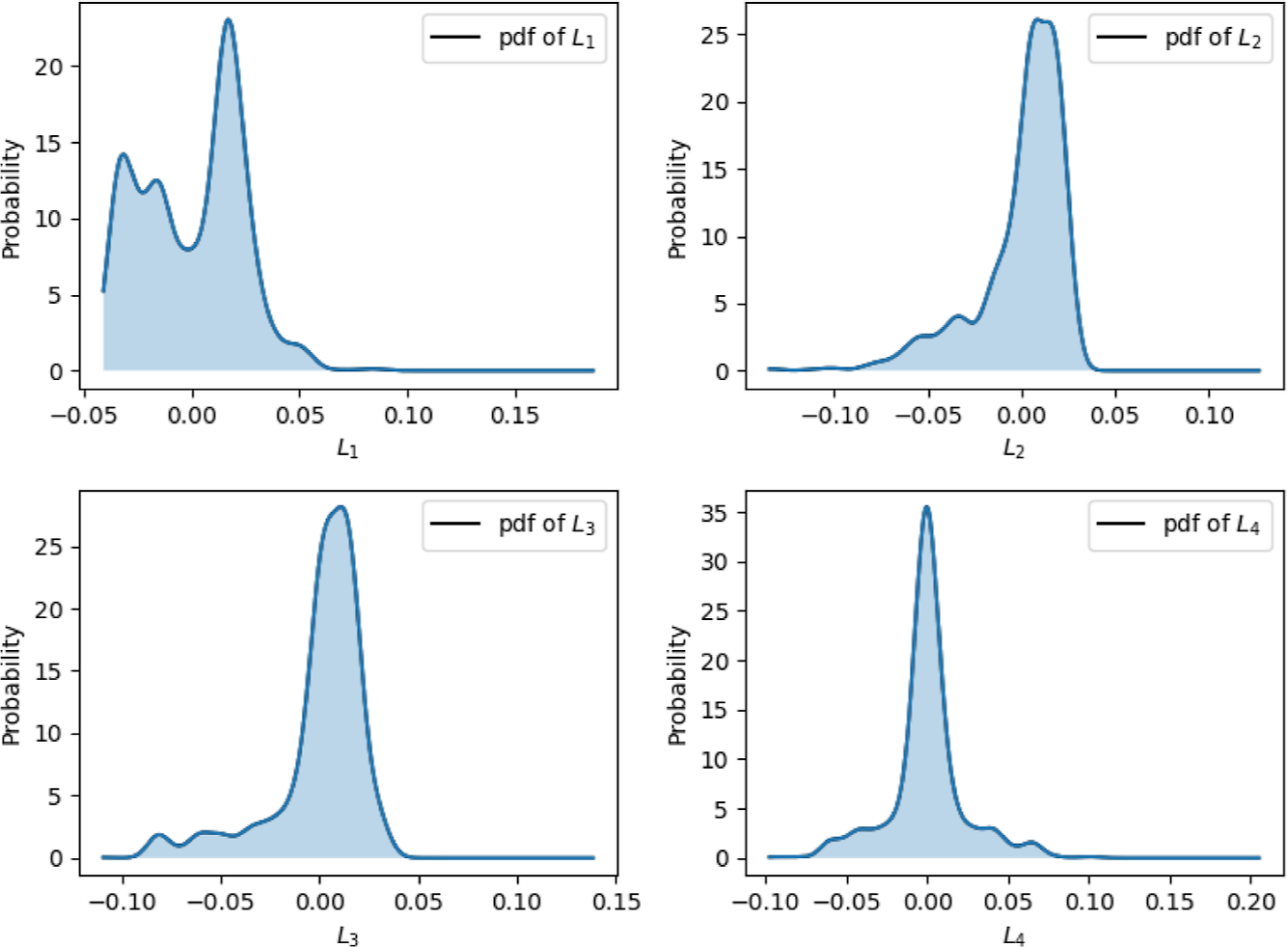
Probability density functions of the LLE representation for the perovskite material.

Once we had our LLE model (4 components, 60 neighbors), we used the *t*-SNE algorithm to see the results of this 4D LLE-transformed space. We ran the *t*-SNE five times in the different cross-validation folds to confirm that these visualizations were consistent. The material groups were the same across all runs, which proves that our 4D LLE space accurately captures the real structure of the data. Materials with MA or FA consistently aggregated into a distinct, elongated cluster, whereas materials comprising MA, Pb, and I (excluding FA or cesium) manifested as a string-like manifold (refer to Supporting Information).

Consider the example of representing the material Cs0.05FA0.81MA0.14PbBr0.45I2.55. First, we obtain the vector **q** (eq 1) given by

$$\begin{array}{lllllllllllll} & Cs & & FA & & MA & & Pb & & Br & & I & \\ {}[\cdot \cdot \cdot & 0.05 & \cdot \cdot \cdot & 0.81 & \cdot \cdot \cdot & 0.14 & \cdot \cdot \cdot & 1 & \cdot \cdot \cdot & 0.45 & \cdot \cdot \cdot & 2.55 & \cdot \cdot \cdot ] \end{array}$$

Which correspond to a sparse vector whose entries are mainly zero. Then, the vector is scaled to unit sum by dividing each element by the sum of all elements. Finally, we apply the LLE transformation just obtained such that we now have a 4th-dimensional vector representing that particular perovskite material (**L** = [0.0165, 0.0174, 0.00068, −0.00424]). This point is represented in Figure 2 alongside with other materials derived from changing the proportions of MA and FA. From that figure we observe a change in the FA-MA proportions is reflected in the *L*_1_ and *L*_2_ dimensions, mainly.

**Figure 2:**
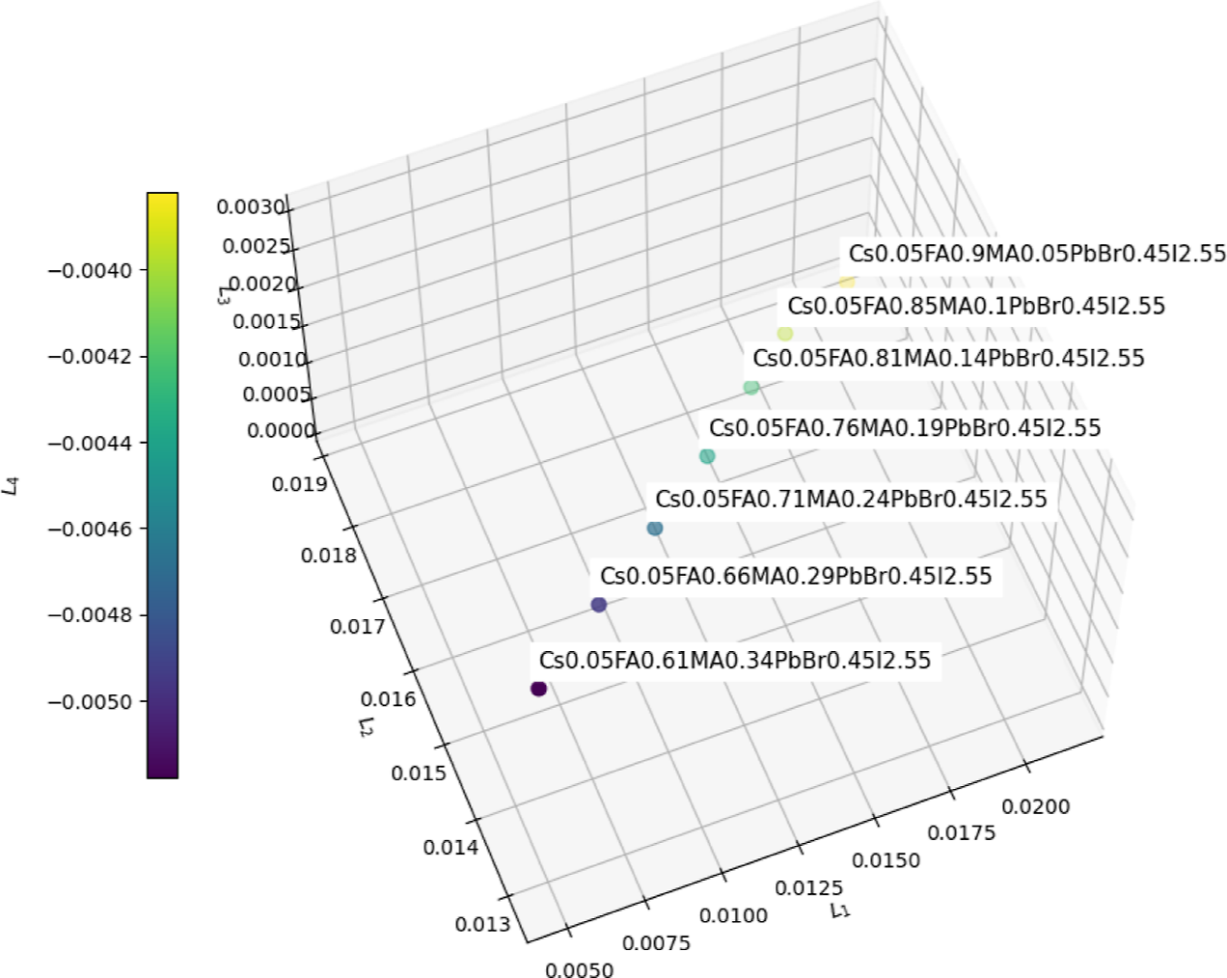
Low-dimensional embedding of perovskite materials using Locally Linear Embedding (LLE = [*L*_1_,*L*_2_,*L*_3_,*L*_4_]). Each point corresponds to a unique composition of the form Cs_0.05_FA_α_MA_β_PbBr_0.45_I_2.55_, where the FA and MA proportions vary. The first three LLE components are plotted in 3D space, and the fourth component is represented by color.

### Synthesis Descriptors

The Perovskite database data set is both quite heterogenoious and sparce, and is thus challenging to work with. In the analysis we have therefore limit ourselves to a smaller number of commonly reported features and reduced the data set accordingly. The focus has been on spin-coated perovskite using a DMF/DMSO solvent mixture which is a type of cells where data has been reported by a large number of papers. In addition to the perovsktie composition, represented by the recently introduced LLE vector, the following continuous synthesis variables were also considered:

- χ_sol._. This feature corresponds to the DMSO/DMF ratio,^20^ where DMSO and DMF (along with other solvents reported in the Perovskite Project Database) are used in the deposition of the perovskite layer. In our case, however, this ratio is expressed in logarithmic form: $\chi_{sol.}=\log_{10}\left(\frac{DMF}{DMSO}\right)$ . But, we added a small constant ϵ = 0.0001 to the denominator for numerical stability.
- *T*_1_. Temperature used during the first thermal annealing stage.^34^
- TB. The thermal budget represents the total heat energy a material is exposed to during thermal processing. We define it as $TB=\log_{10}\left(\sum_{i}t_{i}\cdot T_{i}\right)$ , where *t*_*i*_ and *T*_*i*_ denote the duration and temperature of processing step *i*, respectively.
-
A . Cell area measured refers to the effective area of the device exposed to illumination during performance measurements.
- *E*_g_, Perovskite band gap.

In the present work, as in Lu et al., 2023,^20^ we focus on perovskite solar cell (PSC) devices with power conversion efficiencies (PCE) greater than 10%. Observations containing missing values or non-numerical entries in any variable were excluded from the data set. Additionally, outliers—defined as values with low probability according to their estimated probability density functions—were removed. In particular, we retained only those observations satisfying the following criteria: *V*_oc_ > 0.6 *V*, *E*_g_ > 1.1 eV, $J_{sc}>10\frac{mA}{cm^{2}}$ , and *A* < 5 cm^2^. Once these filters were applied, we obtained a data set of 5441 observations. Figure 3 shows the estimated probability density functions (p.d.f) for these variables, illustrating their underlying distributions. The p.d.f were estimated by using Kernel Smoothing method.

**Figure 3:**
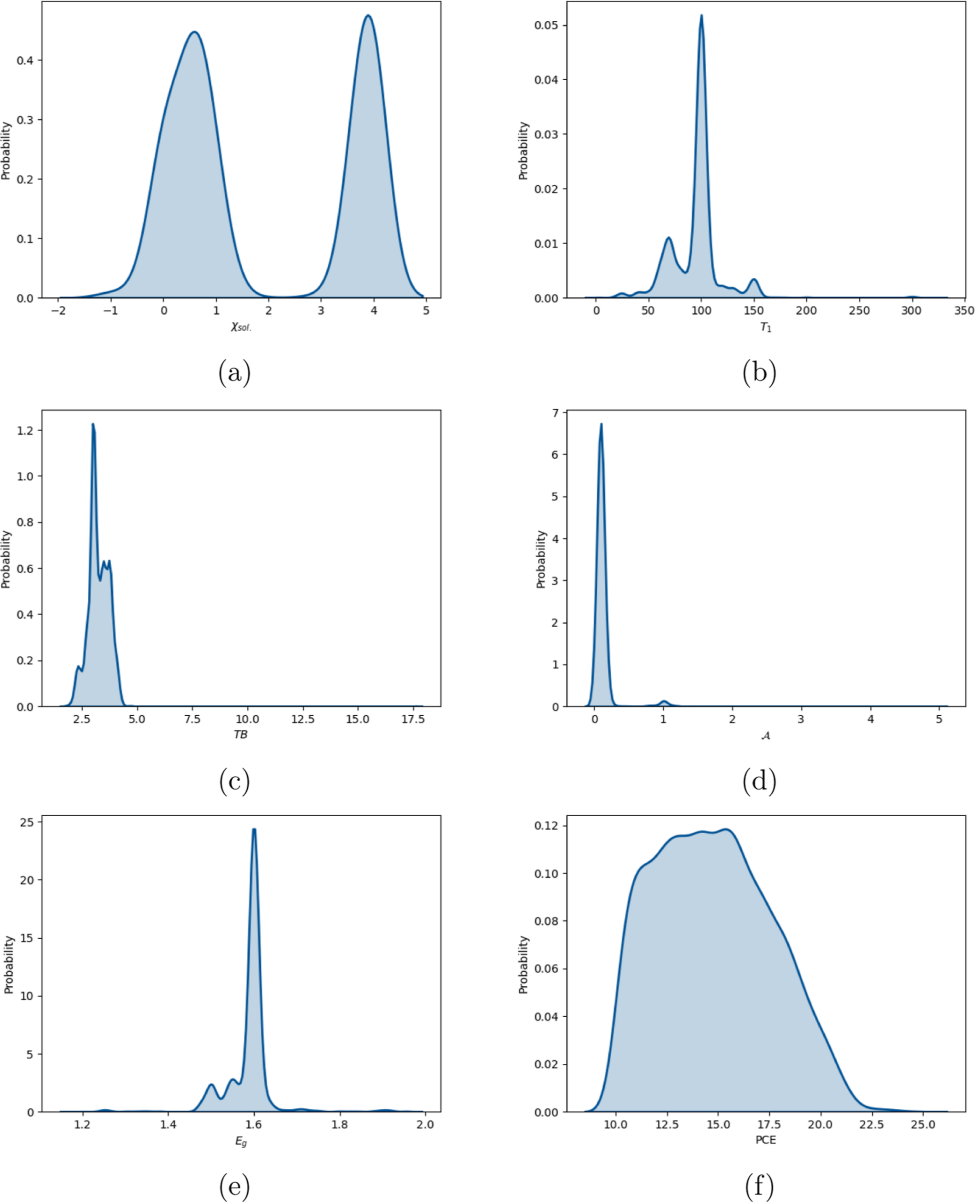
Estimated probability density functions of synthesis variables involved in the probabilistic modeling of PSCs. (a) estimated distribution of χ_sol._, the ratio of DMSO/DMF. (b) distribution of *T*_1_, annealing temperature in the first step. (c) estimated distribution of TB, thermal budget. (d) A , cell area measured. (e) *E*_g_, perovskite band gap. (f) PCE, power conversion efficiency.

### Probability Model

Gaussian Mixture Models (GMMs) are parametric generative models used in unsupervised learning for density estimation and clustering. Since they depict the data as a weighted sum of Gaussian components, each of which encapsulates unique modes or clusters within the data set, they are particularly helpful for modeling multimodal distributions. Examples of multimodal functions are illustrated in Figure 3a,b, which have two and three modes, respectively. GMMs estimate the underlying probability density function of observed data without the need for labeled outputs, in contrast to Gaussian Processes, which are supervised and nonparametric models intended for function approximation. Because of this, GMMs are especially useful in applications where modeling the process’s underlying probabilistic behavior—rather than predicting a particular outcome—is the goal.

The goal is to estimate a probability density function describing the probabilistic behavior of data **Z** = (**X**, *Y*). In order to obtain a probability density model, a data set $Z_{N\times d}$ is required, *N* being the number of observations and *d* the dimension of each observation. Assume we have *d* = *m* + *n* random variables *X*_1_, *X*_2_, ..., *X*_*m*_, *Y*_1_, ..., *Y*_*n*_, which form a random vector **Z** (**z** is used to show a possible value of a random vector **Z**). *X*_1_, *X*_2_, ..., *X*_*m*_ correspond to the *m* = 9 input descriptors or features described in the data section; and, in this particular experiment, *n* = 1 because we are focused on *Y*_1_ = PCE.

The probability density function (pdf) *f*_**Z**_(**z**) = *f*(**z**) can be represented by a linear combination of *J* Gaussian components of dimension *d* as follows

2 $$P\left(z\right)=\sum_{j=1}^{J}\alpha_{j}\cdot N\left(z;\mu^{\left(j\right)},C^{\left(j\right)}\right)$$

where, $N\left(z;\mu^{\left(j\right)},C^{\left(j\right)}\right)$ is a normal joint probability density function with mean **μ** ^(*j*)^ and covariance matrix $C^{\left(j\right)}$ . **μ** ^(*j*)^ is the *j*th *d*-dimensional vector of the *j*th Gaussian component; and, each $C^{\left(j\right)}$ is a matrix of dimension *d* × *d*. In addition, 0 ≤ α_*j*_ ≤ 1 with $\sum_{j=1}^{J}\alpha_{j}=1$ . The expression in (2) is our model.

Regarding model fitting, maximum likelihood is most frequently applied for training probabilistic models. With this approach, the functional form of the probability distribution shown by eq 2 is assumed, and the parameters are changed iteratively by a particular optimization algorithm in order to maximize the likelihood or probability of the observed data, given the suggested model structure.

The likelihood cost function is as follows

3 $$\begin{array}{ll} L\left(\cdot \right) & =\log P\left(\left[z_{1},z_{2},...,z_{N}\right];\mu^{\left(j\right)},C^{\left(j\right)},\alpha_{j}\right)\\ & =\sum_{n=1}^{N}\log\left[\sum_{j=1}^{J}\alpha_{j}N\left(z_{n};\mu^{\left(j\right)},C^{\left(j\right)}\right)\right] \end{array}$$

where, we estimate the parameters of the model (2), $\theta =\left[\alpha_{j},\mu^{\left(j\right)},C^{\left(j\right)}\right]$ for *j* = 1, ...*J*, from data observations [**z**_**1**_, **z**_**2**_, ..., **z**_**N**_]. However, instead of directly maximizing L(θ) , a surrogate function *Q*(θ), satisfying Q(θ)≤L(θ) , is maximized. This approach leads to the Expectation-Maximization (EM) algorithm.^49^

### GMM Regression

Once we have the distribution, we are able to perform tasks such as regression; this means our goal is to estimate the value of the variable *y* = *z*_*p*_, whether we see it as an input or an output since this is an unsupervised method, based on the known variables **x** = [*z*_1_···*z*_*p*–1_ *z*_*p*+1_···*z*_*d*_]. By using the joint probability density function P(z)=P(x,y) of eq 2 we can obtain $P_{Y|X}\left(y|x\right)$ and, finally we estimate the value $ŷ=E\left[Y|X=x\right]=\int yP_{Y|X}\left(y|x\right)dy$ .

### Data Generation

Samples from a GMM, joint as well conditional, can be easily drawn by implementing ancestral sampling. This is a two-step generative procedure that directly follows the model’s mathematical definition. First, a latent component *k* is selected by sampling from a categorical distribution defined by the mixture weights. Second, given the chosen component *k*, a new data point *x* is drawn from its associated multivariate Gaussian distribution $P\left(x|k\right)=N\left(\mu_{k},\Sigma_{k}\right)$ . This process is repeated up to obtaining the desired number of samples.

### Inverse Synthesis Design

Perovskite solar cell design is a many-to-one mapping, meaning many experimental conditions can lead to the same performance. Thus, given a target PCE, if the solution exist, there may be more than one (possibly an infinite) set of input configurations that correspond to it. The many-to-one nature of the direct mapping (conditions → performance) implies the inverse mapping (performance → conditions) is one-to-many, which inherently causes nonuniqueness. When solving this inverse synthesis problem, by using the assistance of the direct problem, this nonuniqueness manifests as a nonconvex cost function with numerous local optima. We propose a generative approach to solve this problem by providing “intelligent starting points” *X*_0_ located in high-potential regions. A Gaussian Mixture Model (GMM) is trained to learn the data’s underlying probability distribution. We then use this GMM to sample promising initial points for the optimization, effectively guiding it toward valid solutions. The complete algorithm is depicted in Figure 4. Standard optimization is precise but gets trapped in local minima, while generative models can explore the whole data landscape but are imprecise. Our method combines their strengths.

**Figure 4:**
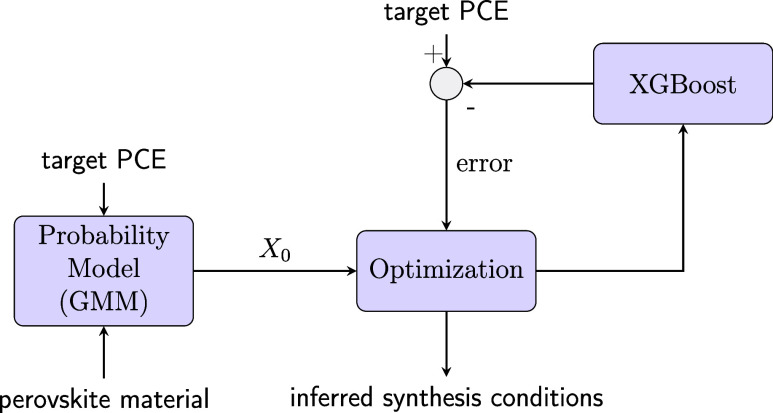
Inverse synthesis design approach to infer the experimental conditions for obtaining the target PCE assuming a particular material. The GMM uses the desired PCE and material properties to generate an initial guess (*X*_0_) for the optimization loop.

In particular, our inverse modeling algorithm works by coupling a generative model (GMM) with a predictive one (XGBoost). The process begins by providing the algorithm with two inputs: the desired target PCE and the material. The GMM uses these inputs to create a conditional probability function of synthesis parameters for that specific case. From this, we sample three initial guesses (*X*_0_) to seed the optimization. Each guess is then refined by the optimization algorithm, which has the goal of minimizing the squared error between the target PCE and the output from the learned machine learning model. Once the three initial guesses have been optimized, the algorithm retains the best-performing experimental configuration. In our case, we selected the optimization method in accordance with the ML model; that is, given that our direct model corresponds to XGBoost, we selected a free-derivative optimization algorithm (e.g., COBYLA^50^).

### Training with Missing Data

Missing data correspond to values that are not seen but would be useful for analysis if they were seen.^51^ This loss of information can make the estimates from machine learning models less accurate. Also, if observations with missing values are systematically different from those with complete data, the remaining complete cases will not be a reliable representative of the original population, making the estimates biased.^52,53^ In addition, missing data could make it harder to model from data because most machine learning methods assume we have full data sets.^54^

Although several databases have been constructed, they could have significant levels of missing information.^18^ There are several approaches to mitigate the missing data problem. The simplest approach is elimination of observations containing missing data.^55,56^ Although this technique is simple and straightforward to implement, its disadvantage lies in the potential reduction of the sample size and the possible resulting bias in subsequent analyses.^56^ In particular, features with the highest number of missing values were eliminated in (Graniero et al., 2023).^34^ This process can be viewed as a preprocessing step.^33^ Another option is to replace missing data with values such as the mean or median of the corresponding variable.^56–58^

Gaussian Mixture Models (GMMs) can be trained even with missing data, eliminating the need to discard incomplete observations. However, this approach requires modifying the Expectation-Maximization (EM) algorithm. In this paper we employ the Expectation Conditional Maximization (ECM) algorithm, originally proposed by^59^ and subsequently utilized by (McCaw et al., 2022)^60^ for modeling by using Gaussian Mixture Models (GMMs) from data sets containing missing data. Unlike complete case analysis, which discards partially observed data, ECM utilizes all available information. The details of the ECM algorithm are available in the Supporting Information accompanying (McCaw et al., 2022).^60^ The GMM model has been adjusted by using a library in the **R** programming language https://CRAN.R-project.org/package=MGMM.

## Results

### Regression

To evaluate the effectiveness of prediction tasks, by using a data set of *N* testing observations, the Root Mean Square Error (RMSE) and the Mean Absolute Error (MAE) are commonly used, with MAE bing a more natural and less ambiguous measure than RMSE.^61^ In addition, RMSE is more penalized than MAE by large errors and outliers. On the other hand we have MAPE (Mean Absolute Percentage Error), that offers a very intuitive interpretation in terms of relative error; but, in real world applications, MAPE can only be used when the quantity to predict is known to remain way above zero.^62^ In addition, MAPE allows for effective comparison of model accuracy and is especially useful when dealing with variables with different units and scales. MAPE is defined as $\frac{1}{N}\sum_{i=1}^{N}\frac{\left|y_{i}-{ŷ}_{i}\right|}{y_{i}}$ , where, *y*_*i*_ and ${ŷ}_{i}$ are the actual value and the predicted value, respectively.

The data set (5441 devices) was randomly split into a development set (80%) and a test set (20%). A 10-fold cross-validation was used exclusively on the development set for training and hyperparameter tuning. We employed the *1-standard-error (1-SE)* rule for hyperparameter tuning to select the optimal number of components. This method selects the simplest model (i.e., the fewest number of clusters) whose cross-validation error is no more than one standard error above the error of the best-performing model. This approach favors a more parsimonious model, resulting in 5 Normal full distributions. The final performance of the model was calculated using the reserved test set (20%), as reported in Table 1. These values are very similar to the obtained in cross-validation process. We compare these results in respect to an XGBoost model that was developed using an ensemble of 50 decision trees. The complexity of each individual tree was constrained to a maximum depth of 7 levels; and, a learning rate of 0.3 was set. The model was then validated by carrying out equivalent training and testing processes.

**Table 1:** Comparison of Regression Performance Metrics (RMSE, MAE, and MAPE) between the Probabilistic GMM Approach and the Discriminative XGBoost Model for PCE Estimated^a^

	RMSE	MAE	MAPE
GMM regression	2.52	2.05	0.14
XGBoost	2.04	1.61	0.12

a While XGBoost achieves superior precision, the GMM maintains competitive accuracy while enabling the generative capabilities.

Focusing on the critical high-efficiency regime (PCE >17%), we observed that the GMM regression accuracy varies significantly between the two identified scenarios. While the first subset exhibits higher deviation (MAE = 3.12, RMSE = 3.41), the second subset demonstrates superior precision, with the MAE and RMSE dropping to 1.73 and 2.18, respectively. This boost in performance is corroborated by the MAPE, which falls from 0.17 to 0.14.

According to recent literature, most successful approaches for predicting PCE are ensemble learning techniques such as XGBoost. In particular, Kasuma et al. (2025), who employed the same original data set used in this study but with different input descriptors, report RMSE = 2.868 and MAE = 1.901 for the XGBoost algorithm, with similar values of RMSE = 2.873 and MAE = 1.899 in the case of Random Forest. Those results are similar to the ones obtained in the present work in the case of GMM regression; however, when using the same input descriptors the XGBoost clearly outperforms the proposed GMM generative approach, as observed in Table 1. It is important to take into account that estimating probability density functions is a considerably more complex task than the standard regression task. It should be noted that estimating discriminative models is easier than explicitly estimating the joint probability distribution;^63^ thus, generative models typically perform worse on regression tasks.

### Discovering Clusters

Cluster discovery could be a useful tool for analyzing the highly complex problem of perovskite solar cell synthesis based on data describing its process. Typically, data is assumed to be a mixture of several distinct groups (instead of one single group), and GMMs automatically segment the data into those clusters that best explain the overall structure. Furthermore, as a generative and probabilistic model, it not only clusters the data, but also describes the underlying distribution that generated it, thus offering a deeper understanding.

Our analysis identified five main clusters (see Figure 5), two of which stand out for their contrasting performance profiles (blue and red). The lowest-performing cluster (in red) is characterized by a centroid with the highest values for the thermal budget, the highest band gap (*E*_g_ = 1.81 eV), and its particularly high first annealing temperature value (*T*_1_ = 139 °C), as observed in Table 2. The model isolated a distinct failure mode (1.5% of samples) characterized by high values in thermal budget TB, *E*_g_, and *T*_1_, which consistently resulted in the worst-performing devices. On the other hand, the second worst-performing cluster (in orange) is characterized by extreme value in its solvent ratio χ_sol._. The best cluster center is characterized by a moderate value on its thermal budget, a band gap around 1.56, a solvent ratio of about 9.5 parts of DMF for each part of DMSO, and a first annealing temperature of about 102 °C.

**Figure 5:**
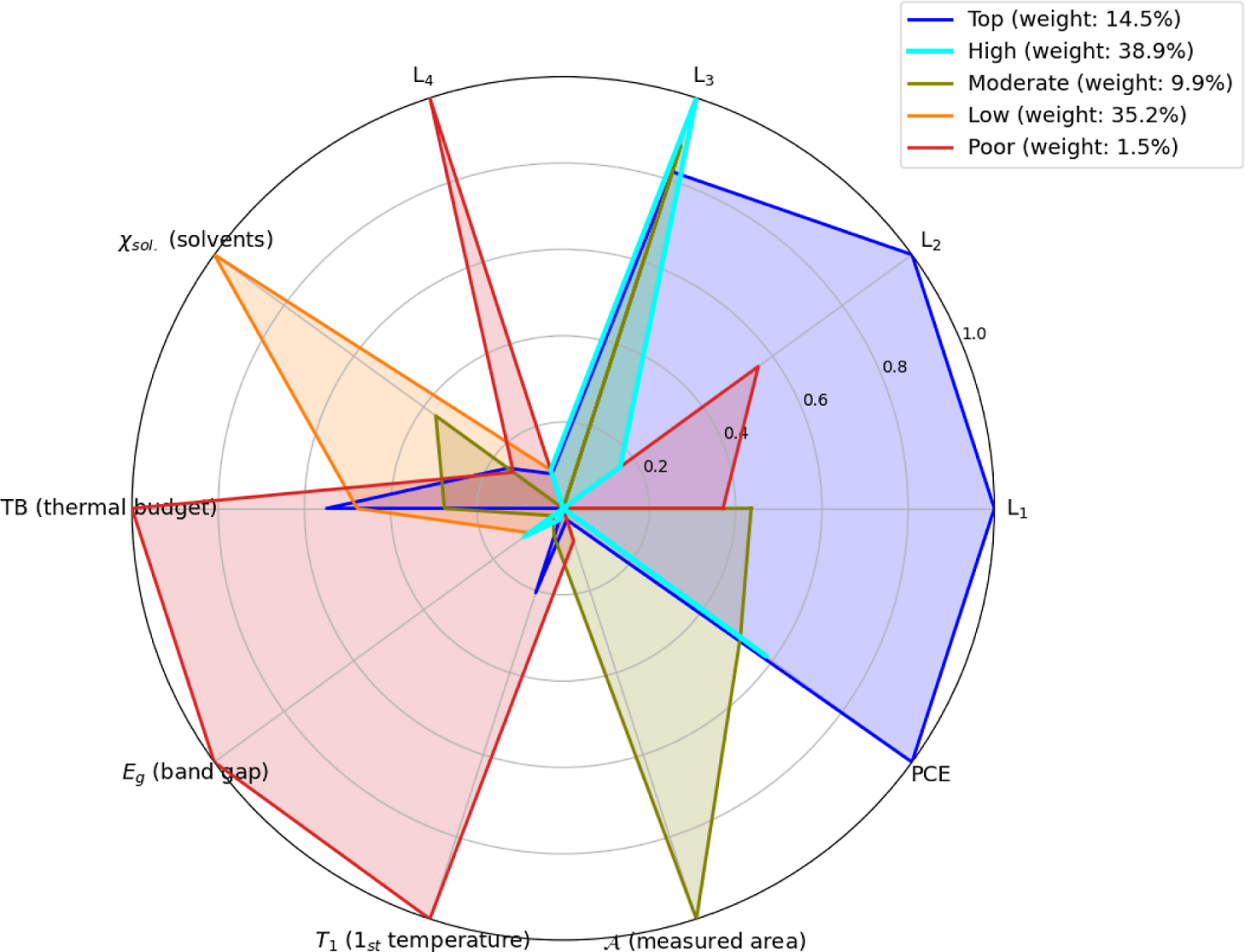
Radar plot visualizing the cluster centers (prototypes) identified by the Gaussian Mixture Model (GMM). Each colored polygon represents the normalized mean profile for one of the seven clusters, showing its value for 10 key features. The features include those describing the materials (*L*_1_, *L*_2_, *L*_3_, *L*_4_), process variables, and the final device performance (PCE).

**Table 2:** Centroids (Mean Vectors) of the 5 Components in the Final Trained Gaussian Mixture Model^a^

no.	*L*_1_	*L*_2_	*L*_3_	*L*_4_	χ_sol._ (solvents)	TB (thermal budget)	*E*_g_ (band gap)	*T*_1_ (1st temp.)	A (area)	PCE
0	0.020725	0.020448	0.004198	–0.001907	9.52	2473.70	1.56	101.95	0.10	16.62
1	–0.036007	0.006587	0.017516	–0.001388	2.69	1136.00	1.59	93.60	0.09	15.05
2	–0.011275	0.003875	0.008882	–0.006726	49.27	1678.21	1.57	95.51	0.47	14.77
3	–0.036007	0.006587	0.017516	–0.001389	too high	2236.31	1.59	92.33	0.10	13.76
4	–0.014976	0.013148	–0.056429	0.050345	8.37	4693.82	1.81	139.13	0.12	12.86

a Each row represents the center of a discovered data cluster, showing the archetypal experimental conditions and the corresponding mean PCE for that group.

### Data Generation

We obtain 1051 observations, the same number of observations in the testing set, and we assess the ability of our probabilistic model to generate new samples. A good generation method should preserve the statistical properties of the original data; therefore, we compare the data distributions between the generated data and the original data. In the present work we use the energy distance test of homogeneity^64^ in order to analyze if two samples (e.g., real vs generated data) are drawn from the same underlying probability distribution. This test is a nonparametric method not requiring any assumption about the data’s underlying distribution; it is able to reliably compare multivariate data and not just for 1D data like classical statistical tests. Furthermore, it avoids the need for hyperparameter tuning like kernel-based methods. The null hypothesis corresponds to H_0_: random vectors have the same distribution, for which high values in its statistic are evidence that favors the alternative hypothesis H_1_: random vectors are drawn from different distributions.

In both cases, we obtain *p*-values =0.01, the minimum possible when selecting 99 for the number of resamples to take in the permutation test. That is, with a confidence level greater than 95%, we can affirm that the estimated distributions, for both cases, are different from the original.

A key advantage of GMMs is their ability to compute conditional probabilities, which results in being useful for inverse design. To illustrate this, Figure 6 presents the 2D conditional probability contours for the χ_sol._ (solvent ratio) and TB variables, assuming the perovskite material is MAPbI_3_. The plots compare two distinct scenarios: assuming we have a target PCE of 14% (left) and a target PCE of 18% (right).

**Figure 6:**
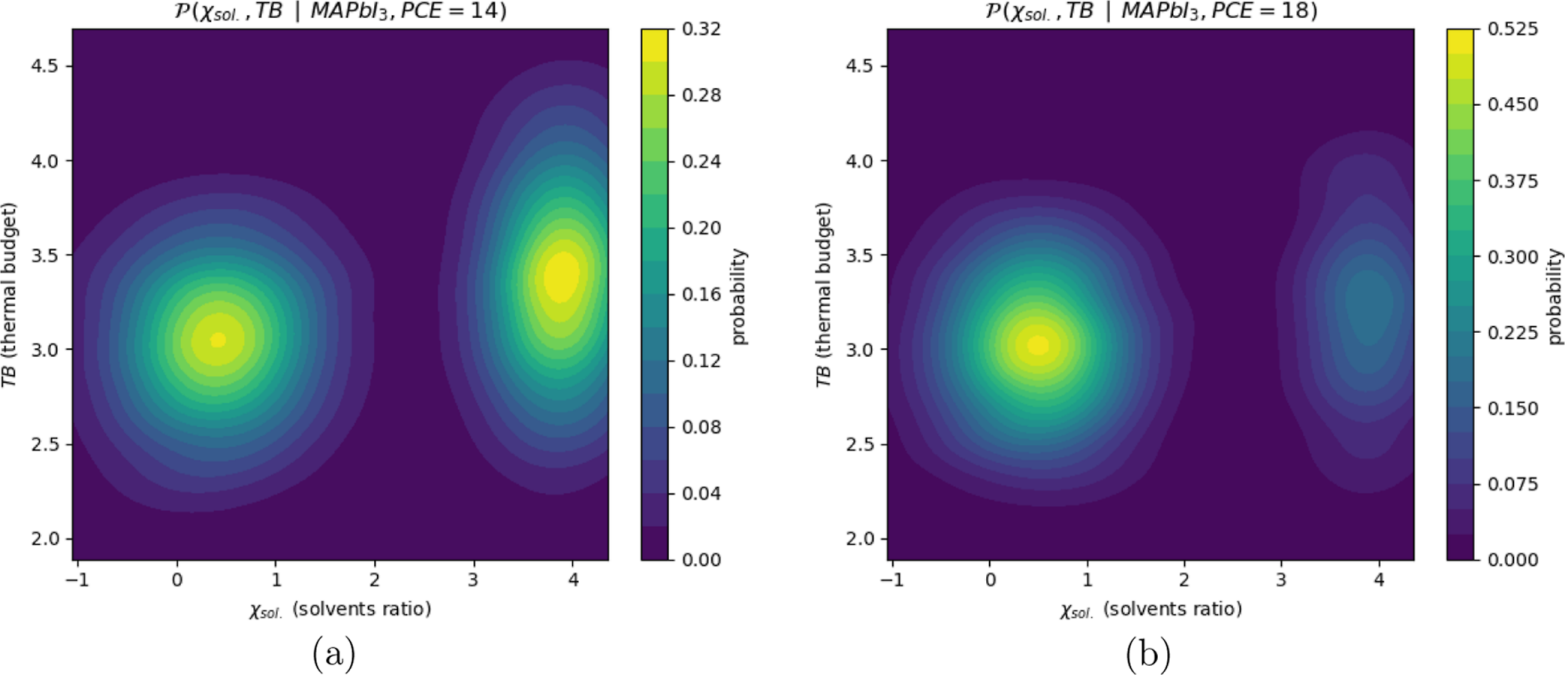
Estimated joint probability density functions of DMF/DMSO ratio and annealing thermal budget for two synthetic data sets representing different solar cell efficiencies. Left: $P\left(\chi_{sol.},TB|MAPbI_{3},PCE=14\right)$ exhibits a bimodal distribution. Right: $P\left(\chi_{sol.},TB|MAPbI_{3},PCE=18\right)$ shows a single dominant mode.

These conditional plots provide critical insights. The plot corresponding to 18% PCE reveals that while a moderate solvent ratio is the most probable path to high performance, a secondary, viable but less common region exists at high DMF-to-DMSO ratios. This highlights the “many-to-one” nature of the problem. Conversely, the 14% PCE plot (left) serves as a caution; that it, having that moderate solvent ratios do not guarantee success and can still result in moderately low PCEs.

### Inverse Synthesis Modeling

Consider the following scenario: Given prior knowledge about the perovskite material (MAPbI_3_) and assuming a target PCE, one may seek to identify the set of synthesis conditions most likely to yield the desired performance. In this context, conditional probability provides a framework for modeling the distribution of synthesis parameters conditioned on specified criteria, enabling data-driven exploration of inverse design scenarios. We compare our GMM-assisted optimization method with conventional random-start optimization for inverse design tasks. Three starting points were randomly selected in both cases. Then, the material and process “recipes” generated by both methods were then fed back into a pretrained forward XGBoost model to get the corresponding “predicted PCE”, and the nearest result to the target PCE was retained.

The target-predicted PCE pairs were plotted to visualize how closely each method’s results were in respect to the “perfect coincidence” line. The plot’s results clearly demonstrate the superior performance of the GMM-assisted optimization (blue circles in Figure 7). These points follow in a better way the “perfect coincidence” line, proving that this method consistently finds material recipes that achieve the desired target PCE, or at least a very close one. In sharp contrast, the random-start optimization (gray squares in Figure 7) shows significant scattering and a large performance gap, especially for higher targets. Calculating the RMSE reveals the superior performance of the GMM-assisted optimization, which achieved a value of 1.52, in sharp contrast to the 3.32 from the random-start method.

**Figure 7:**
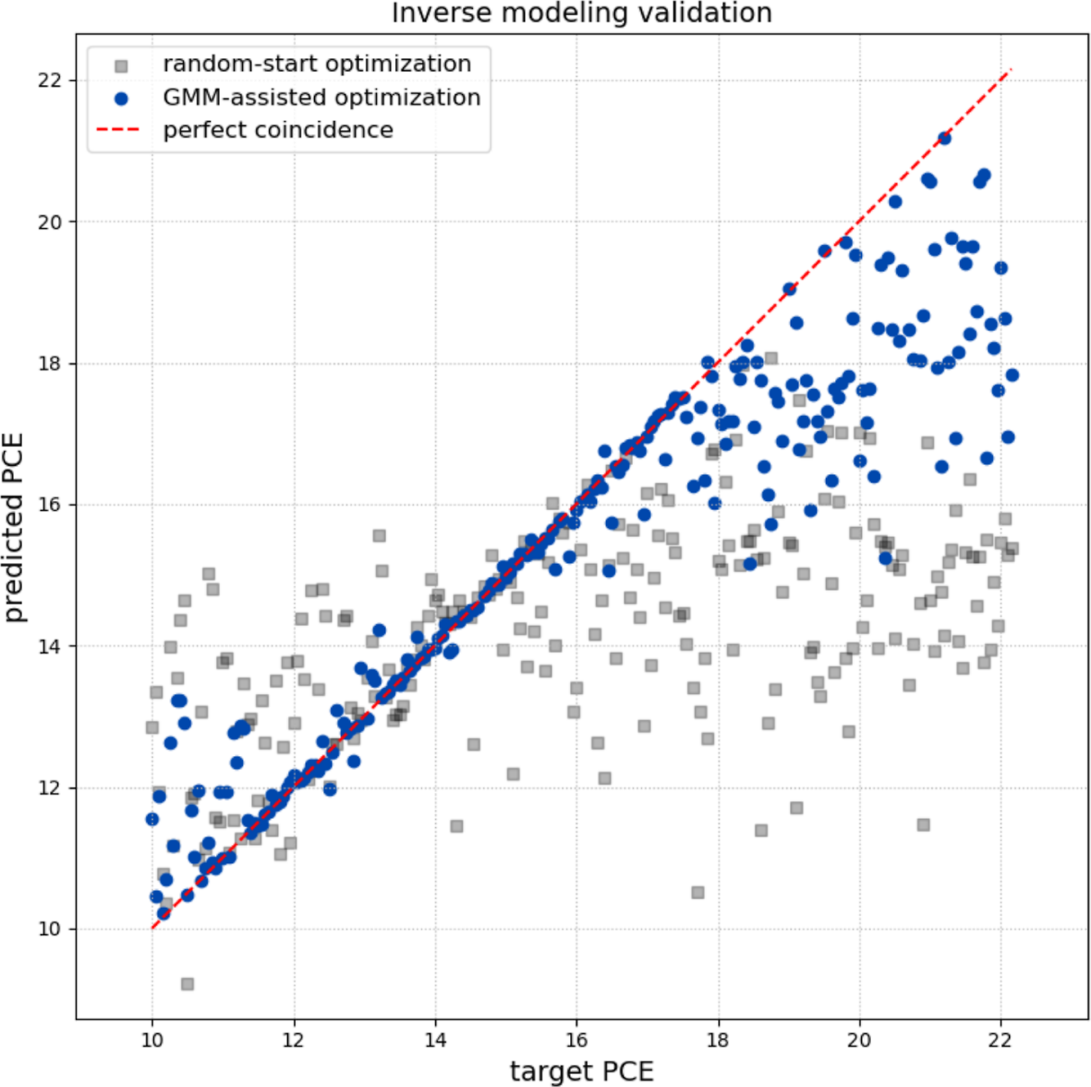
Validation of the Inverse Synthesis Modeling Algorithm. Each gray point represents a distinct inferred configuration, showing its predicted PCE against the corresponding target. The dashed line indicates perfect correlation.

The results show a stronger agreement in the range from 13% to approximately 17% for the case of the GMM-assisted algorithm. However, above 18%, the predicted PCEs begin to “saturate” and fall off the target line. The result indicates that even when we ask for a target of 20%, the optimizer fails to find inputs that can achieve it. This is not a failure of the optimizer. It suggests that for the given material (MAPbI3), the XGBoost model has learned that a PCE higher than ∼ 18.5% is physically unattainable, regardless of the synthesis conditions.

### Modeling with Missing Data

To evaluate the proposed method, we begin with the complete data set described above and artificially introduce missing values. MAPE metrics for different missing data levels (5%, 10%, 15%, 20%) are estimated by using *K*-fold cross-validation (*K* = 5). The PCE performance prediction is assessed in the following scenarios: mean data imputation (GMM + mean); median data imputation (GMM + median); list-wise deletion (GMM without observaions with missing entries); and GMM trained with ECM (MGMM). The results of these scenarios are depicted in Figure 8.

**Figure 8:**
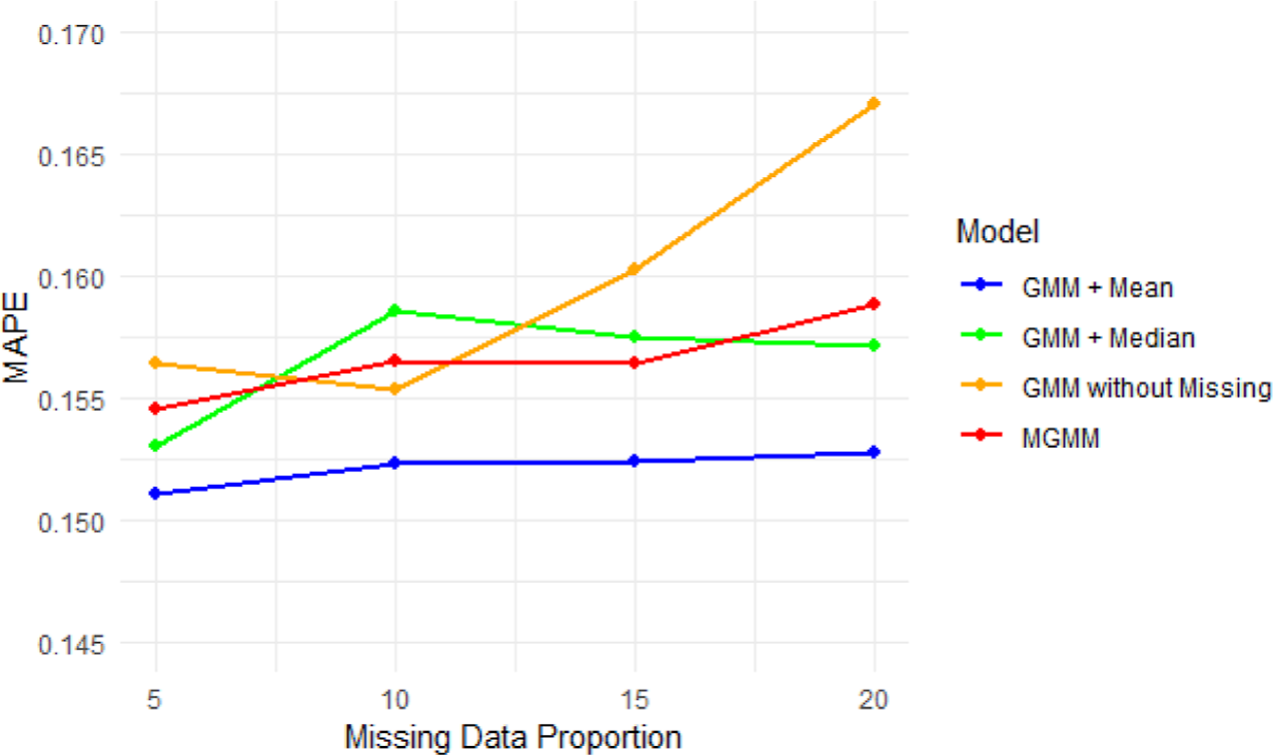
Performance comparison of different GMM-based models under increasing proportions of missing data, MAPE (Figure 8). The models include GMM with mean and median imputation, GMM with listwise deletion (removing observations with missing entries), and MGMM.

The model relying on listwise deletion shows a noticeable decline in performance as missingness increases, especially at 20% missing data. Reducing the effective sample size by discarding incomplete observations considerably affects the model’s performance. In contrast, MGMM maintains relatively stable error levels across all metrics and missingness proportions, outperforming listwise deletion in higher missingness scenarios. MGMM does not obtain the lowest error, but it maintains stability without having to rely on arbitrary imputation. Thus, it could be seen as an alternative and scalable solution for real-world scenarios with incomplete data, although its performance is comparable with mean and median imputation.

## Conclusions and Future Work

While much research focuses on discriminative models, we have shown that a generative GMM is an interpretable and statistically sound choice for the scarce and low-to-medium-dimensional tabular data common in materials science. In this work, we have successfully demonstrated that a unified probabilistic framework based on a Gaussian Mixture Model (GMM) is a pragmatic and versatile tool for analyzing perovskite solar cell data. We have validated this framework’s utility across five distinct tasks: discovering hidden clusters, performing regression, generating novel candidates, and model learning in missing data scenarios. This confirms that a single, well-chosen model can serve as a comprehensive, analytical, and versatile tool for experimental data sets.

The most significant contribution of this work is our novel “GMM-Assisted Optimization” method for inverse synthesis design. By using the GMM’s conditional probability to identify high-potential starting points, we provide an intelligent “seed” for the local, derivative-free exploitation optimizer. This work provides a new, robust, and highly efficient method for accelerating the data-driven discovery of high-performance perovskite solar cells and offers a framework that could be broadly applied to other challenges in materials science.

Regarding future directions, we propose two key ones. As data set sizes increase, the generative component should evolve from GMMs to deep learning approaches, such as tabular diffusion models, to capture high-dimensional complexities and then generate seed candidates. Second, from an optimization perspective, replacing the tree-based forward predictor (XGBoost) with a differentiable surrogate model (e.g., a neural network) would be advantageous. This would make it possible to calculate gradients (first- and second-order derivatives), accelerating convergence and improving accuracy even more.

## Supplementary Material



## Data Availability

The complete data set has been downloaded form the Perovskite Database Project at https://www.perovskitedatabase.com/; and, the curated data set used for the analysis is available at 10.5281/zenodo.16809654. The code used for running the analysis and for generating the figures are available at https://github.com/alexander-sepulveda/pdf_prvskts.git.

## Footnotes

1
https://www.nrel.gov/pv/cell-efficiency.

## Notes

Additional details about the workflow of the experiment can be found in the enclosed file. The Supporting Information is available free of charge at https://pubs.acs.org/doi/10.1021/acs.jcim.5c02017.

t-SNE of LLE components; model fitting; GMM regression; illustrative example; reproduction of experiment (PDF)
