# Supplementary material for: Density Estimation Based on Mixtures of Gaussians for Perovskite Solar Cells Modeling
Source: J Chem Inf Model. 2026 Jan 19;66(3):1371–83. doi: 10.1021/acs.jcim.5c02017 (PMC12892318; doi:10.1021/acs.jcim.5c02017)
Supplement: Supplementary file 1 [file ci5c02017_si_001.pdf]

Density estimation based on mixtures of Gaussians for perovskite solar cells modeling: supporting information

Alexander Sepúlveda,*,† Daniel Cerro,† and T. Jesper Jacobsson*,‡

†*School of Electrical, Electronics and Telecommunications Engineering, Universidad Industrial de Santander, Bucaramanga-Colombia.*

‡*Department of Physics, Chemistry and Biology (IFM), Linköping University, Linköping, Sweden*

E-mail: alexander.sepulveda@saber.uis.edu.co; jesper.jacobsson@liu.se

t-SNE of LLE components

We implemented t-Distributed Stochastic Neighbor Embedding (t-SNE) to project the 4-dimensional manifold obtained via Locally Linear Embedding (LLE) for the sake of verifying the effectiveness of the dimensionality reduction process. See figures (1) and (2). We observe that the reduction from the original material representation vectors to a compact latent space successfully preserves the intrinsic structure.

Model fitting

There are several steps in the modeling process, where the selection of the model structure (i.e. the kind of model) is one of the first. This involves defining a family of models, usually of infinite quantity, from which the best-fitting model is inferred. Then, a training criterion

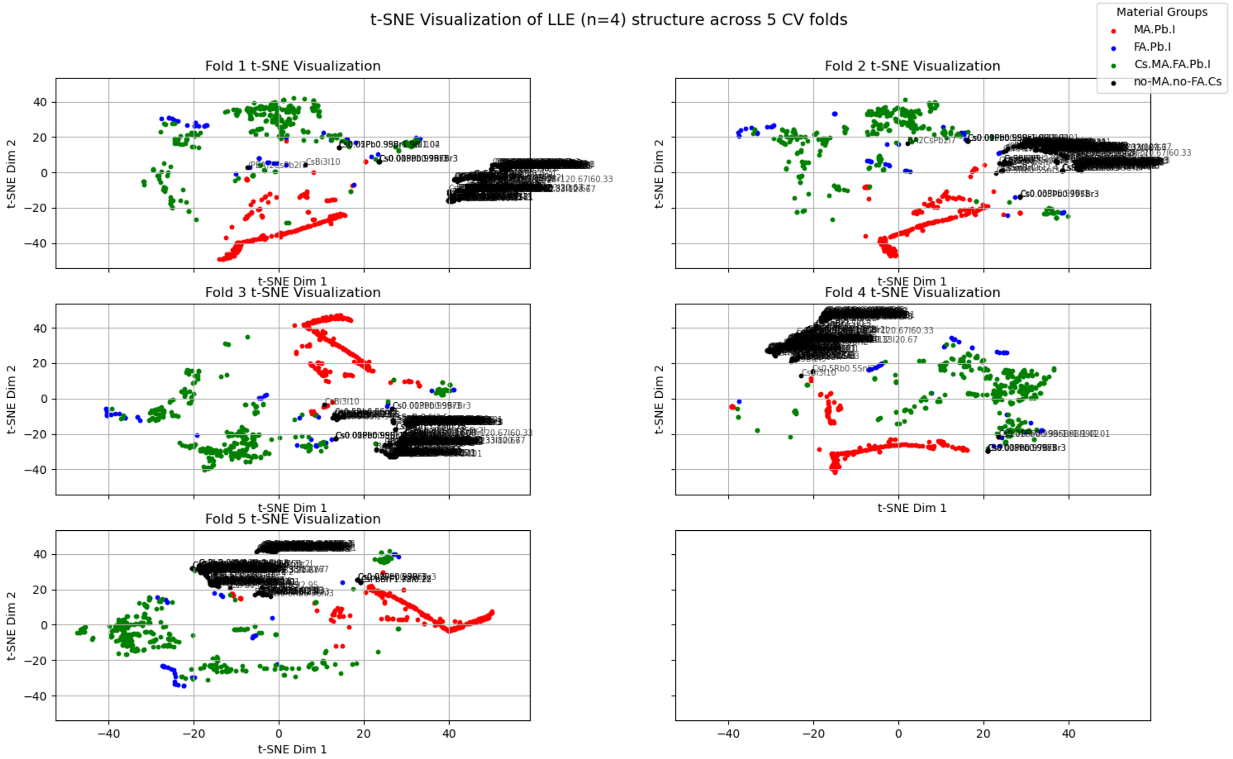

Figure 1: t-SNE representation of Locally Linear Embedding components ($LLE = [L_1, L_2, L_3, L_4]$) for each fold in the crossvalidation procedure.

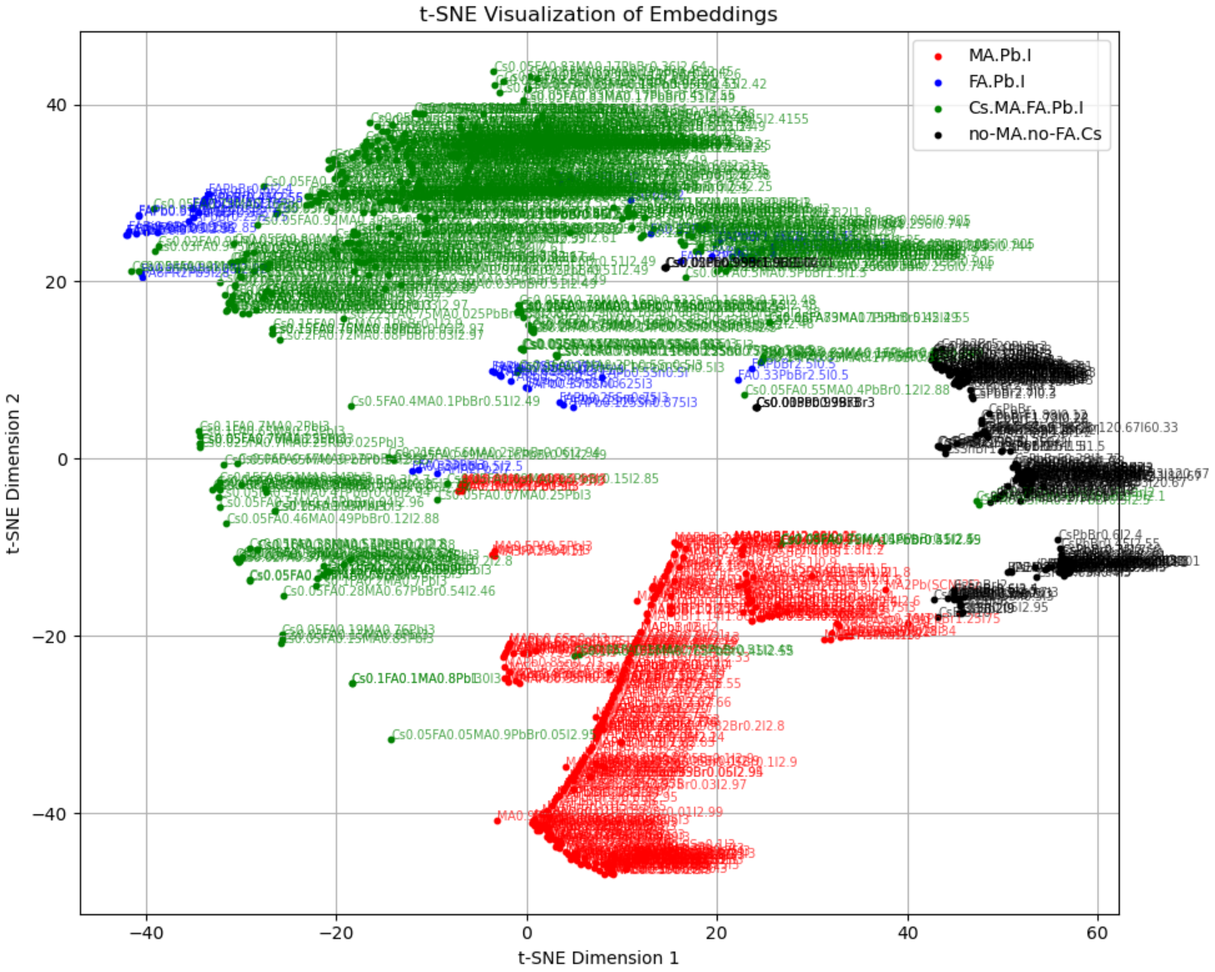

Figure 2: t-SNE representation of Locally Linear Embedding components ($LLE = [L_1, L_2, L_3, L_4]$) for the dataset.

for estimating the parameters of the model that best fits the data should be selected.

In general, maximum likelihood (ML), maximum a posteriori (MAP), and minimum mean squared error (MMSE) are the three main criteria for estimating the parameters. Among them, maximum likelihood is most frequently applied for training probabilistic models. With this approach, the functional form of the probability distribution, given by weighted sum of multivariate Gaussians, is assumed; and, the parameters are changed iteratively by a particular optimization algorithm in order to maximize the likelihood or probability of the observed data, given the suggested model structure.

We estimate the parameters of the model GMM model, $\theta = [\alpha_j, \boldsymbol{\mu}^{(j)}, \mathbb{C}^{(j)}]$ for $j = 1, \dots J$, from data observations $[\mathbf{z_1}, \mathbf{z_2}, \dots, \mathbf{z_N}]$. Each example $\mathbf{z}_j$ from the set of training observations is evaluated in the model (**??**), obtaining the probability $\mathcal{P}(\mathbf{z}_j)$. Then, given that we assume the observations $\mathbf{z}_j$ are statistically independent between them, the overall probability for the entire training set $\mathcal{P}\big(\cdot\big)$ is calculated as the multiplication of all those probabilities $\mathcal{P}(\mathbf{z}_j)$. However, that value would be extremely small; thus, the logarithm function is included in the cost function, transforming the multiplication operation into a sum. The likelihood cost function is as follows:

$$\begin{aligned}\mathcal{L}\big(\cdot\big) &= \log \mathcal{P}\Big([\mathbf{z_1}, \mathbf{z_2}, \dots, \mathbf{z_N}]; \boldsymbol{\mu}^{(j)},\, \mathbb{C}^{(j)},\, \alpha_j\Big) \\ &= \sum_{n=1}^{N} \log \left[\sum_{j=1}^{J} \alpha_j \mathcal{N}\Big(\mathbf{z_n}; \boldsymbol{\mu}^{(j)}, \mathbb{C}^{(j)}\Big)\right]\end{aligned} \tag{1}$$

However, instead of directly maximizing $\mathcal{L}(\theta)$, a surrogate function $Q(\theta)$, satisfying $Q(\theta) \leq \mathcal{L}(\theta)$, is maximized. This approach leads to the *Expectation-Maximization* (EM) algorithm.[?]

GMM Regression

Once we have the distribution, we are able to perform tasks such as *regression*; this means our goal is to estimate the value of the variable $y = z_p$, whether we see it as an input or an output

since this is an unsupervised method, based on the known variables $\mathbf{x} = [z_1 \cdots z_{p-1} z_{p+1} \cdots z_d]$. We use the conditional expected value,

$$\widehat{y} = E\big[Y \mid \mathbf{X} = \mathbf{x}\big] = \int y \mathcal{P}_{Y|\mathbf{X}}(y \mid \mathbf{x}) dy \tag{2}$$

then, using the joint probability density function $\mathcal{P}(\mathbf{z}) = \mathcal{P}(\mathbf{x}, y)$ of equation (**??**) we can obtain $f_{Y|\mathbf{X}}(y \mid \mathbf{x})$ and, finally we estimate the value $\hat{y}$ by using (2).

For each j_{th} component we take the covariance matrices $\mathbb{C}^{(j)}_{\mathbf{X}}$, $C^{(j)}_{Y\mathbf{X}}$ from $\mathbb{C}^{(j)}$, as shown in equation (3). In our case we have 9 descriptors and 1 output (the PCE) so $d = 10$. The matrix $\mathbb{C}^{(j)}_{\mathbf{X}}$ is the 9×9 covariance matrix of available measurements; $\mathbf{C}^{(j)}_{Y\mathbf{X}}$ is a 1×9 vector; and, $\mathbf{C}^{(j)}_{\mathbf{X}Y}$ (the transpose of $\mathbf{C}^{(j)}_{Y\mathbf{X}}$) is a 9×1 column vector. In addition, $C^{(j)}_{Y} = \text{var}(Y)$ (the variance of Y).

$$\mathbb{C}^{(j)} = \left[\begin{array}{c:c} \mathbb{C}^{(j)}_{\mathbf{X}} & \mathbf{C}^{(j)}_{\mathbf{X}Y} \\ & \\ \hdashline \mathbf{C}^{(j)}_{Y\mathbf{X}} & C^{(j)}_{Y} \end{array}\right] \tag{3}$$

On the other hand $\boldsymbol{\mu}^{(j)} = [\boldsymbol{\mu}^{(j)}_X \vdots \boldsymbol{\mu}^{(j)}_Y]^\top$.

The estimated value $\hat{y}$ is obtained by,

$$\hat{y} = E\big[Y \mid \mathbf{X} = \mathbf{x}\big] = \sum_{j=1}^{J} \beta_j(\mathbf{x}) \cdot m_j(\mathbf{x}) \tag{4}$$

where,

$$\beta_j = \frac{\alpha_j \cdot \mathcal{N}(\mathbf{x}; \mu^{(j)}_{\mathbf{X}}, C^{(j)}_{\mathbf{X}})}{\sum_{j=1}^{J} \alpha_j \cdot \mathcal{N}(\mathbf{x}; \mu^{(j)}_{\mathbf{X}}, C^{(j)}_{\mathbf{X}})} \tag{5}$$

The expressions β_j (that is $\mathcal{P}(j \mid \mathbf{X} = \mathbf{x})$) are also called responsabilities.[?] The numerator of β_j corresponds to the probability obtained by evaluating the value $\mathbf{x}$ in the j_{th} Gaussian component. The same value, $\mathbf{x}$, is evaluated in all the mixture components, and the results are added. This sum acts as a normalization factor, allowing β_j to be interpreted as the proportion—or "responsibility"—that component j has in generating the data $\mathbf{x}$.

On the other hand, the expression $m_j(\mathbf{x})$ in (6) corresponds to the conditional mean given $\mathbf{x}$ in respect to the multivariate gaussian component j. It is as follows,

$$m_j(\mathbf{x}) = \mu_Y^{(j)} + \mathbf{C}_{Y\mathbf{X}}^{(j)} \cdot \mathrm{inv}(\mathbb{C}_{\mathbf{X}}^{(j)}) \cdot (\mathbf{x} - \mu_{\mathbf{X}}^{(j)}) \tag{6}$$

As shown in (4), the estimate $\hat{y}$ is computed as a weighted sum of m_j, the estimates provided by each Gaussian component. The appendix shows the detailed steps to these equations.

Illustrative example

To illustrate how GMMs can capture the probabilistic behavior, we consider a simplified experiment. We consider the same dataset of 5441 observations used in §(GMM Regression); but, in this example, the input variable is the perovskite band gap, while the output variable is the short-circuit current density (J_{sc}); and the probabilistic model is the bi-dimensional joint probability distribution $\mathcal{P}(E_g,\, J_{sc})$ represented by a GMM. Real-world data often exhibits multimodal behavior, where different physical regimes or fabrication conditions lead to distinct clusters in the data.

In particular, in this illustrative example $\mathbf{X} = E_g$ and $Y = J_{sc}$ so $\mathbf{Z} = [E_g, J_{sc}]$. The dimension of the problem is $d = m+n = 2$, with $m = 1$ and $n = 1$. In addition, based on the scatter plot of figure (3(a)), we decided to use $J = 2$ two gaussian components. The K-means algorithm was employed to initialize the centroids for the EM algorithm. The probability density function is then estimated, obtaining the following explicit mathematical expression:

$$\begin{aligned} \mathcal{P}(z) = \alpha_1 \cdot \frac{1}{2\pi} \cdot \det(\mathbb{C}^{(1)})^{-1/2} \cdot \exp\left(-\frac{1}{2}(\boldsymbol{z} - \boldsymbol{\mu}^{(1)})^\top \cdot (\mathbb{C}^{(1)})^{-1} \cdot (\boldsymbol{z} - \boldsymbol{\mu}^{(1)})\right) \\ + \alpha_2 \cdot \frac{1}{2\pi} \cdot \det(\mathbb{C}^{(2)})^{-1/2} \cdot \exp\left(-\frac{1}{2}(\boldsymbol{z} - \boldsymbol{\mu}^{(2)})^\top \cdot (\mathbb{C}^{(2)})^{-1} \cdot (\boldsymbol{z} - \boldsymbol{\mu}^{(2)})\right) \end{aligned} \tag{7}$$

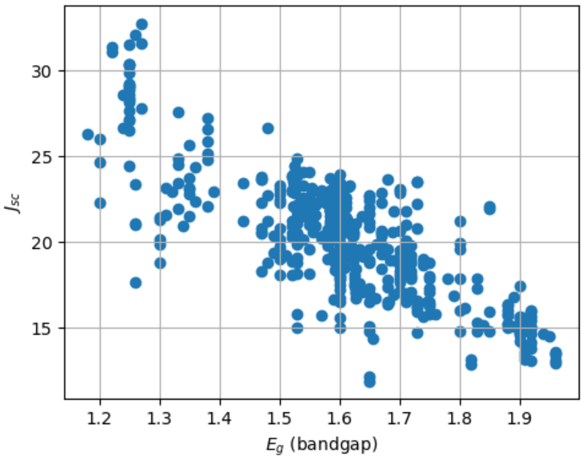

(a) Scatter plot of E_g versus J_{sc}

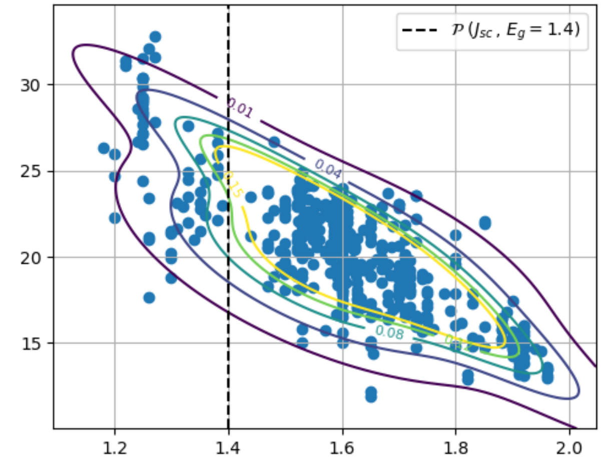

(b) estimated probability density function contours.

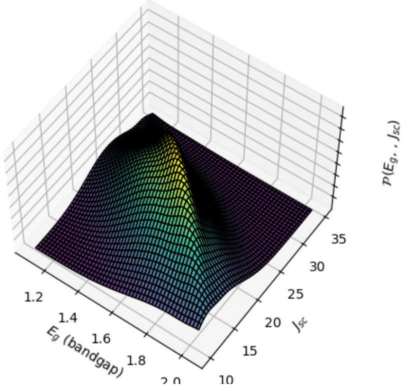

(c) 3D plot of the estimated probability density function.

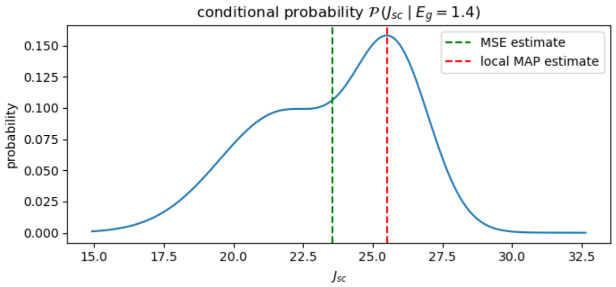

(d) J_{sc} prediction assuming $E_g = 1.4$ by using MSE and MAP criteria.

Figure 3: Illustrative example for the estimation of J_{sc} from E_g by using a estimated probability density function based on GMMs. Figure 3a presents a scatter plot of the data; Figure 3b shows the estimated probability density function (p.d.f.); Figure 3c displays the same p.d.f. in a 3D representation; and Figure 3d illustrates the MSE and MAP estimates.

where,

$$\alpha_1 = 0.51, \quad \boldsymbol{\mu}^{(1)} = \begin{bmatrix} 1.59 \\ 19.73 \end{bmatrix}, \quad \mathbb{C}^{(1)} = \begin{bmatrix} 0.024 & -0.262 \\ -0.262 & 8.194 \end{bmatrix}$$

$$\alpha_2 = 0.49, \quad \boldsymbol{\mu}^{(2)} = \begin{bmatrix} 1.63 \\ 20.77 \end{bmatrix}, \quad \mathbb{C}^{(2)} = \begin{bmatrix} 0.036 & -0.799 \\ -0.799 & 19.334 \end{bmatrix}$$

The resulting pdf expression (7) is depicted figure (3)(b-c). Figure (3)(b) shows the estimated pdf contours, where the dashed line corresponds to the joint probability function $\mathcal{P}(J_{sc},\, E_g = 1.4)$; and, figure (3)(c) shows the joint pdf in three dimensions. Having the joint probability $\mathcal{P}(J_{sc},\, E_g)$ we are now able to calculate $E\big[J_{sc} \mid E_g\big]$ (the conditional expected value of J_{sc} given E_g). For the case of $E_g = 1.4$ The conditional probability $\mathcal{P}(J_{sc} \mid E_g = 1.4)$ is depicted in figure (3)(d). It also depicts the estimate of J_{sc} given $E_g = 1.4$, using two different estimation criteria: minimum mean squared error (MMSE) and maximum-a-posteriori (MAP). The MMSE estimate tends to smooth over the multimodal structure, while the MAP criterion selects the point with the highest probability. In this illustrative example we implemented the local MAP (nearest local MAP) instead of the MAP; that is, the mode of the gaussian that is closest to a given reference point is selected, rather than choosing the global maximum of the conditional posterior probability function.

Now, we are going to calculate the MMSE estimate by using equation (4) given the model (7). First, we evaluate the point $x = E_g = 1.4$, obtaining $\mathcal{N}_1 = 1.235$ and $\mathcal{N}_2 = 1.034$. Then, we calculate $\beta_j(x = 1.4)$; $j = 1, 2$ ($\beta_1 = 0.56$, $\beta_2 = 0.44$). Next, we calculate $m_j(x = 1.4)$; $j = 1, 2$ ($m_1 = 21.82$, $m_2 = 25.75$). Finally, we obtain $E\big[J_{sc} \mid E_g = 1.4\big] = 0.56 \cdot 21.82 + 0.44 \cdot 25.75 = 23.6$. This value is shown in green in Figure 3(d). It should be interpreted as follows: for an observed band gap of 1.4 eV, the expected short-circuit current (the mean with respect to the resulting density function) is 23.6 $\frac{\text{mA}}{\text{cm}^2}$. In contrast, according to the MAP criterion, this means that for an observed band gap of 1.4 eV, the most likely short-circuit current is 25.51 $\frac{\text{mA}}{\text{cm}^2}$.

Reproduction of experiment

The complete dataset has been downloaded from the *Perovskite Database Project* at `https://www.perovskitedatabase.com/`; and, the curated dataset used for the analysis is available at `https://doi.org/10.5281/zenodo.16809654`. The scripts used for running the analysis and for generating the figures are available at `https://github.com/alexander-sepulveda/pdf_prvskts.git`.
